# Digenic sarcomeric variants in paediatric dilated cardiomyopathy and maternal peripartum cardiomyopathy: a familial case report

**DOI:** 10.1093/ehjcr/ytag105

**Published:** 2026-02-12

**Authors:** Hakan Kurt, Zülal Ülger Tutar, Ertürk Levent, Burcugül Karasulu Beci, Eser Doğan

**Affiliations:** Division of Pediatric Cardiology, Department of Pediatrics, Ege University Faculty of Medicine, Kazımdirik, Bornova, 35100, Izmir, Turkey; Division of Pediatric Cardiology, Department of Pediatrics, Ege University Faculty of Medicine, Kazımdirik, Bornova, 35100, Izmir, Turkey; Division of Pediatric Cardiology, Department of Pediatrics, Ege University Faculty of Medicine, Kazımdirik, Bornova, 35100, Izmir, Turkey; Division of Pediatric Cardiology, Department of Pediatrics, Ege University Faculty of Medicine, Kazımdirik, Bornova, 35100, Izmir, Turkey; Division of Pediatric Cardiology, Department of Pediatrics, Ege University Faculty of Medicine, Kazımdirik, Bornova, 35100, Izmir, Turkey

**Keywords:** Left ventricular non-compaction, Dilated cardiomyopathy, Peripartum cardiomyopathy, Myosin heavy chains, Myosin-binding protein C, Case report

## Abstract

**Background:**

Left ventricular non-compaction (LVNC) can be observed as a phenotypic trait in patients with dilated cardiomyopathy. Familial cases have been increasingly recognized, with sarcomeric gene mutations—particularly in *MYH7* and *MYBPC3*—playing a significant role. Peripartum cardiomyopathy (PPCM) may also share overlapping genetic architecture with inherited cardiomyopathies.

**Case summary:**

We report a 7-year-old girl with a clinical diagnosis of dilated cardiomyopathy with LVNC since infancy. Genetic analysis revealed two heterozygous missense variants in sarcomeric genes associated with inherited cardiomyopathies: *MYH7*: c.4186C>T (p.Arg1396Trp) and *MYBPC3*: c.2672G>A (p.Arg891Gln), both classified as variants of uncertain significance. Segregation analysis showed that the *MYH7* variant was maternally inherited and the *MYBPC3* variant paternally inherited. Notably, the mother developed PPCM 4 months postpartum, with an ejection fraction (EF) of 35%–40%, and was found to carry the same *MYH7* variant. The father remained asymptomatic. This case highlights a potential familial cardiomyopathy syndrome with phenotypic variability: the child presenting with an dilated cardiomyopathy with LVNC phenotype and the mother with PPCM. The presence of distinct cardiomyopathy phenotypes within the same family carrying shared and separate sarcomeric variants suggests a possible genotype–phenotype correlation, emphasizing the importance of comprehensive genetic screening and long-term familial surveillance in such cases.

**Discussion:**

This report highlights the clinical relevance of identifying digenic sarcomeric variants in paediatric cardiomyopathy, particularly when associated with a positive maternal history of PPCM. Familial evaluation and recognition of genotypic overlap may aid in risk stratification and management.

Learning points

- Digenic heterozygous sarcomeric variants may contribute to severe early-onset dilated cardiomyopathy with a left ventricular non-compaction phenotype in paediatric patients.
- Shared sarcomeric gene variants can manifest as distinct cardiomyopathy phenotypes within the same family, including paediatric dilated cardiomyopathy and maternal peripartum cardiomyopathy (PPCM).
- Identification of overlapping genotypes in paediatric cardiomyopathy and PPCM supports the need for comprehensive genetic testing and long-term familial surveillance.

## Introduction

Left ventricular non-compaction (LVNC) is characterized by a prominent trabecular meshwork, a thin compacted layer, and deep intertrabecular recesses.^1^ Left ventricular non-compaction shows incomplete penetrance and variable expressivity, spanning prenatal/neonatal onset to asymptomatic adulthood, and is familial in ∼30% of cases.^2^ Genetically, defects disrupting myocardial compaction—most often in sarcomeric or related genes (*ACTC1*, *MYH7*, *MYBPC3*, *TNNT2*, *TPM1*, *TTN*, *LDB3*, *LMNA*, *DTNA*)—are frequently implicated.^2,3^ Diagnosis relies on imaging; Jenni’s echocardiographic criteria use an end-systolic non compacted/compacted ratio (NC/C ratio) >2, while magnetic resonance imaging (MRI)/computed tomography (CT) studies suggest a diastolic NC/C ratio >2.3 to improve specificity and distinguish pathology from physiologic hypertrabeculation.^4^ Peripartum cardiomyopathy presents with reduced LVEF late in pregnancy or early postpartum and can overlap genetically with familial dilated cardiomyopathy (DCM)/LVNC; familial clustering and DCM-gene variants support selective genetic testing.^5^ Up to 20% of PPCM patients harbour pathogenic variants—most commonly *TTN* truncations—while sarcomeric genes such as *MYH7* and *MYBPC3* also contribute via impaired structure/contractility.^6,7^

## Summary figure

**Figure:**
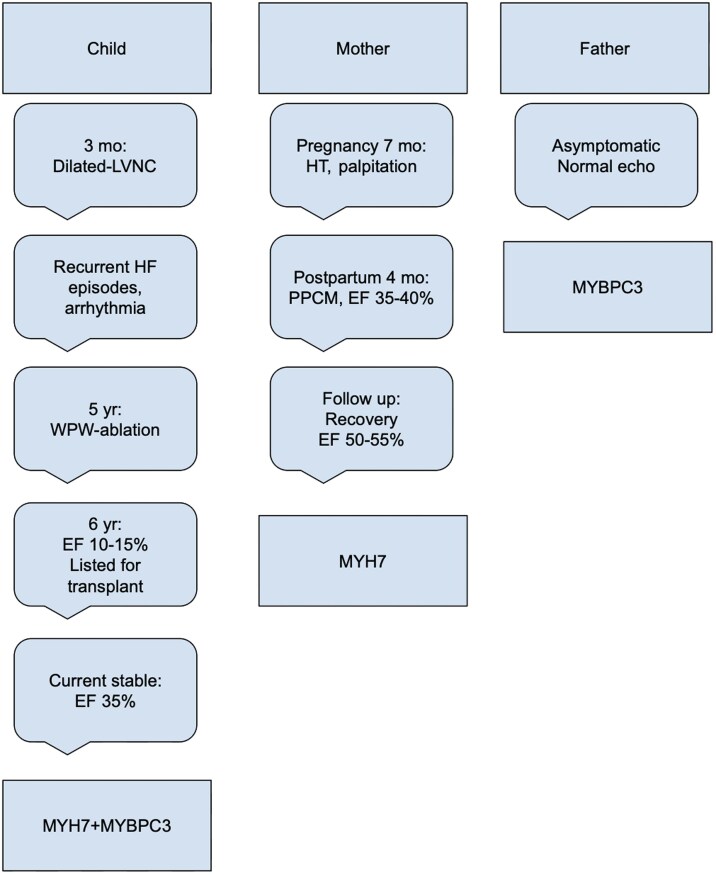


## Case presentation

**Figure 1:**
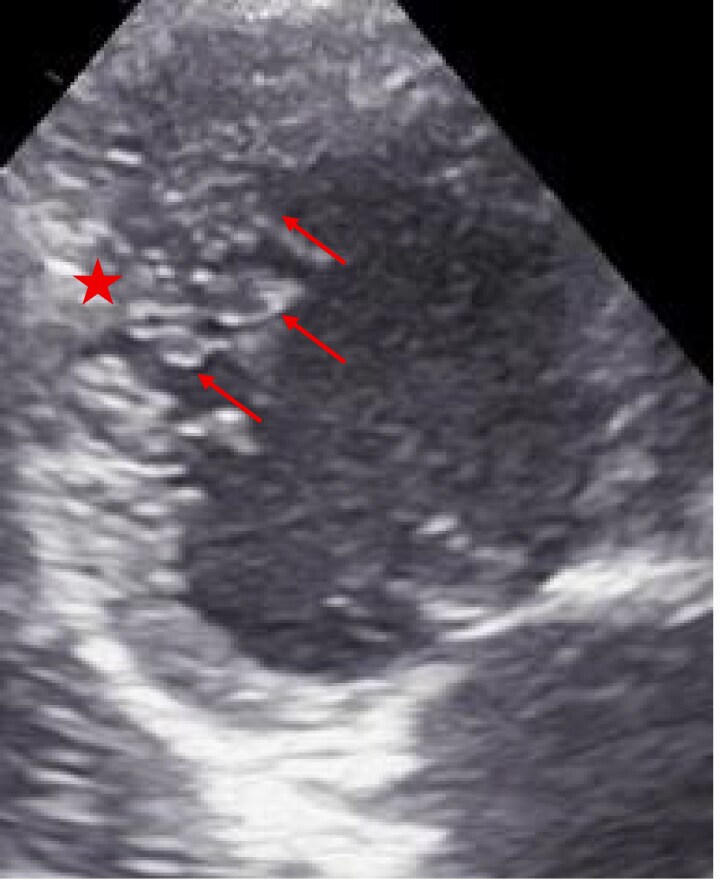
Apical long-axis view from transthoracic echocardiography demonstrating prominent trabeculations and deep intertrabecular recesses within the left ventricular apex, consistent with morphological features of left ventricular non-compaction. Red arrows highlight the regions of prominent trabeculation. The red asterisk indicates the compact myocardial layer.

**Figure 2:**
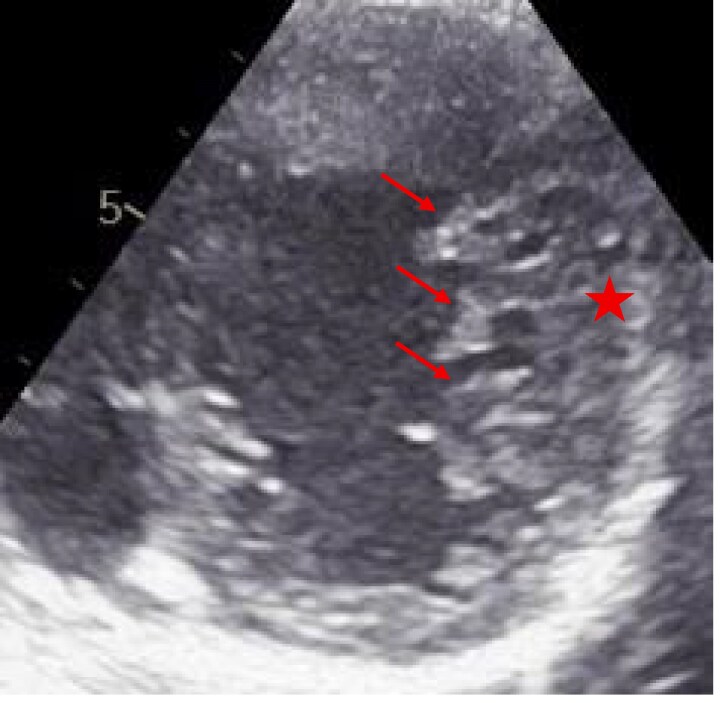
Short-axis echocardiographic view showing excessive trabeculations and deep intertrabecular recesses in the left ventricular myocardium, suggestive of left ventricular non-compaction. Red arrows highlight the regions of prominent trabeculation. The red asterisk indicates the compact myocardial layer.

A 7-year-old girl, diagnosed with DCM with LVNC at 3 months, was born at term by caesarean section with normal perinatal course and age-appropriate neurodevelopment. She experienced recurrent heart failure and supraventricular tachycardia with intermittent Wolff–Parkinson–White; at age 5, electrophysiology confirmed a right posterolateral accessory pathway and successful ablation. At 6 years, transplant evaluation showed prominent LV trabeculation with NC/C ratio 2.6 and LVEF 10%–15% (*Figures 1* and *2*). Work-up was negative for metabolic/infectious aetiologies; CT angiography showed cardiomegaly, biventricular dilation, and increased trabeculation. She was listed for transplantation and closely followed. Targeted next-generation sequencing identified heterozygous missense variants in *MYH7* (NM_000257.4:c.4186C>T, p.Arg1396Trp) and *MYBPC3* (NM_000256.3:c.2672G>A, p.Arg891Gln); segregation showed maternal inheritance of *MYH7* and paternal inheritance of *MYBPC3*. The non-consanguineous mother (30 years) developed gestational hypertension and tachycardia; 4 months postpartum she presented with dyspnoea/palpitations and was diagnosed with PPCM (LVEF 35%–40%); on follow-up, her LVEF improved to 50%–55% with mild global hypokinesia and increased trabeculation (NC/C ratio 1.4). The father (31 years) is asymptomatic with normal echocardiography. Genetic analysis was limited to the parents, as other family members were not accessible or available for testing. The child remains clinically stable on optimized therapy, with LVEF 30%–35%, and is listed for heart transplantation.

## Discussion

Recent genetic studies highlight the role of sarcomeric mutations, particularly in *MYH7* and *MYBPC3*, in LVNC, DCM, and PPCM. Pathogenic variants in these genes are found in about 25% and 13% of LVNC cases, respectively, with a domain-specific correlation: *MYH7* head-domain variants associate with isolated LVNC and fewer adverse events, while tail-domain variants more often link to an LVNC/DCM overlap and may disrupt titin binding, mimicking *TTN* effects.^8^ In a large *MYH7*-related DCM cohort, LVNC features were common across ages, reinforcing the LVNC-DCM overlap.^9^ Although often milder in adult-onset LVNC, *MYH7* mutations can cause severe perinatal disease, as shown in foetuses with hydrops fetalis from maternal mosaicism and in infants with biventricular non-compaction and restrictive physiology, both leading to early heart failure and death.^2,10^ Overall, pathogenic sarcomeric variants—especially compound heterozygous or digenic states—occur in up to 22% of LVNC and are linked to more severe phenotypes, suggesting additive or modifying effects.^3,11^

In another report, digenic inheritance involving *MYH7* and *LAMA4* was linked to severe early-onset DCM, with the *LAMA4* variant acting as a genetic modifier.^12^ Similarly, Petropoulou *et al*.^13^ described a consanguineous family with dual *MYH7* and *TNNT2* variants, where only individuals carrying both mutations exhibited disease, reinforcing the concept of synergistic genetic interactions.

Our paediatric case presents with a severe infant-onset DCM with LVNC phenotype in the setting of dual heterozygous missense variants in *MYH7* (c.4186C>T; p.Arg1396Trp) and *MYBPC3* (c.2672G>A; p.Arg891Gln), inherited maternally and paternally, respectively. The mother, who carries only the *MYH7* variant, was diagnosed with PPCM 4 months postpartum, displaying a milder but clinically significant phenotype with reduced ejection fraction. In contrast, the father—carrier of the *MYBPC3* variant—remains asymptomatic with normal cardiac imaging.

The *MYBPC3* c.2672G>A (p.Arg891Gln) variant is a missense change classified in ClinVar as a Variant of Uncertain Significance (VUS) due to limited evidence and is not listed in OMIM as directly linked to LVNC. Although its pathogenicity is uncertain, *MYBPC3* is a well-established cardiomyopathy gene, particularly in hypertrophic cardiomyopathy (HCM), with some variants also reported in LVNC.

The *MYH7* c.4186C>T (p.Arg1396Trp) variant, likewise listed in ClinVar as a VUS and not individually associated with LVNC or DCM in OMIM, affects the β-myosin heavy chain. While this specific change lacks direct evidence, *MYH7* is extensively implicated in HCM, DCM, and LVNC.

In our patient, the *MYH7* variant is non-truncating and localized to the tail domain, consistent with the LVNC/DCM overlap phenotype. As reported by van Waning *et al*.,^8^ such tail-domain variants may disrupt titin binding and phenocopy the effects of *TTNtv*, conferring a higher risk of systolic dysfunction. These features further support the need for a genotype-guided approach to management and surveillance in cardiomyopathy patients, particularly in the peripartum context.

The early-onset and more severe clinical presentation in our case may reflect a potential synergistic or modifying effect of carrying both variants, supporting a hypothesis of cumulative pathogenicity or digenic interaction. In addition, this familial observation illustrates the need for long-term cardiologic follow-up of the asymptomatic father, given his carrier status of a sarcomeric gene variant (*MYBPC3*) that may yet lead to a late-onset phenotype.

In PPCM, growing evidence supports its overlap with familial DCM, particularly in patients harbouring pathogenic variants in cardiomyopathy-associated genes. Multiple studies have shown that 15%–20% of PPCM patients carry variants in genes such as *TTN*, *MYH7*, *MYBPC3*, *LMNA*, and *SCN5A*. Pregnancy-related stressors may unmask subclinical cardiomyopathy in gene-positive, asymptomatic women, leading to overt heart failure.^14,15^

van Spaendonck-Zwarts *et al*.^5^ conducted a foundational study in families with coexisting PPCM and DCM, revealing pathogenic mutations in over half of the families. Familial PPCM patients showed significantly lower recovery rates of left ventricular function compared to general PPCM populations, suggesting genetic predisposition may confer worse outcomes.

Our case further supports the notion that PPCM may be a manifestation of underlying genetic predisposition, potentially triggered or unmasked by the haemodynamic stresses of pregnancy. The presence of a shared variant between mother and child, manifesting as distinct cardiomyopathy phenotypes, underscores the variable expressivity often seen in sarcomeric gene-related cardiomyopathies.

## Conclusion

This case highlights the complexity of genotype–phenotype relationships in inherited cardiomyopathies and underscores the clinical relevance of digenic sarcomeric variants in early-onset disease. The co-occurrence of paediatric DCM with LVNC phenotype and maternal PPCM within the same family supports a possible role for *MYH7* variants in both phenotypes and raises the need to consider inherited cardiomyopathy even in apparently sporadic cases. These findings emphasize the importance of comprehensive family screening, careful variant interpretation in clinical context, and long-term surveillance of asymptomatic carriers. Moreover, further functional studies are warranted to clarify the pathogenic potential of variants that currently fall below established classification thresholds but may still contribute meaningfully to disease expression.

## Lead author biography


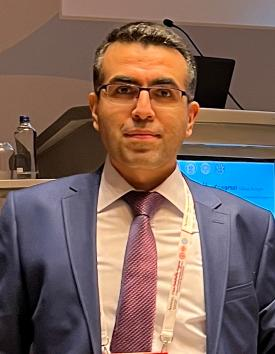
2010–16: Medical Education, Istanbul University, Cerrahpaşa, Faculty of Medicine. 2018–23: Residency in Paediatrics, Mersin University, Department of Pediatrics. June 2024–present: Fellowship in Paediatric Cardiology, Ege University, Division of Pediatric Cardiology

**Consent:** Written informed consent was obtained from the patient’s legal guardians for the publication of this case report and any accompanying images.

## Data Availability

The data underlying this article will be shared on reasonable request to the corresponding author.

## References

[1] Jaouadi H, El Louali F, Wanert C, Cano A, Ovaert C, Zaffran S. Dilated-left ventricular non-compaction cardiomyopathy in a pediatric case with SPEG compound heterozygous variants. Int J Mol Sci 2022;23:5205. 35563595 10.3390/ijms23095205 PMC9102709

[2] Kawamura H, Ikawa M, Hirono K, Kimura J, Okuno T, Kawatani M, et al Low-frequency maternal novel MYH7 mosaicism mutation in recurrent fetal-onset severe left ventricular non-compaction: a case report. Front Pediatr 2023;11:1195222. 37360367 10.3389/fped.2023.1195222 PMC10285293

[3] Sedaghat-Hamedani F, Haas J, Zhu F, Geier C, Kayvanpour E, Liss M, et al Clinical genetics and outcome of left ventricular non-compaction cardiomyopathy. Eur Heart J 2017;38:3449–3460. 29029073 10.1093/eurheartj/ehx545

[4] Yu W, Thomas MA, Mills L, Wright JR Jr. Prenatal diagnosis of isolated right ventricular non-compaction cardiomyopathy with an MYH7 likely pathogenic variant. Fetal Pediatr Pathol 2023;42:464–471. 36630130 10.1080/15513815.2022.2120785

[5] van Spaendonck-Zwarts KY, Posafalvi A, van den Berg MP, Hilfiker-Kleiner D, Bollen IA, Sliwa K, et al Titin gene mutations are common in families with both peripartum cardiomyopathy and dilated cardiomyopathy. Eur Heart J 2014;35:2165–2173. 24558114 10.1093/eurheartj/ehu050

[6] Sliwa K, Bauersachs J, Arany Z, Spracklen TF, Hilfiker-Kleiner D. Peripartum cardiomyopathy: from genetics to management. Eur Heart J 2021;42:3094–3102. 34322694 10.1093/eurheartj/ehab458

[7] Spracklen TF, Chakafana G, Schwartz PJ, Kotta MC, Shaboodien G, Ntusi NAB, et al Genetics of peripartum cardiomyopathy: current knowledge, future directions and clinical implications. Genes (Basel) 2021;12:103. 33467574 10.3390/genes12010103 PMC7830587

[8] van Waning JI, Moesker J, Heijsman D, Boersma E, Majoor-Krakauer D. Systematic review of genotype–phenotype correlations in non-compaction cardiomyopathy. J Am Heart Assoc 2019;8:e012993. 31771441 10.1161/JAHA.119.012993 PMC6912966

[9] de Frutos F, Ochoa JP, Navarro-Peñalver M, Baas A, Bjerre JV, Zorio E, et al Natural history of MYH7-related dilated cardiomyopathy. J Am Coll Cardiol 2022;80:1447–1461. 36007715 10.1016/j.jacc.2022.07.023

[10] Miura F, Shimada J, Kitagawa Y, Otani K, Sato T, Toki T, et al MYH7 mutation identified by next-generation sequencing in three infant siblings with bi-ventricular non-compaction presenting with restrictive hemodynamics. J Cardiol Cases 2019;19:140–143. 30996762 10.1016/j.jccase.2018.12.017 PMC6451088

[11] Hoedemaekers YM, Caliskan K, Michels M, Frohn-Mulder I, van der Smagt JJ, Phefferkorn JE, et al The importance of genetic counseling, DNA diagnostics, and cardiologic family screening in left ventricular non-compaction cardiomyopathy. Circ Cardiovasc Genet 2010;3:232–239. 20530761 10.1161/CIRCGENETICS.109.903898

[12] Abdallah AM, Carlus SJ, Al-Mazroea AH, Alluqmani M, Almohammadi Y, Bhuiyan ZA, et al Digenic inheritance of LAMA4 and MYH7 mutations in patient with infantile dilated cardiomyopathy. Medicina (Kaunas) 2019;55:17. 30650640 10.3390/medicina55010017 PMC6359299

[13] Petropoulou E, Soltani M, Firoozabadi AD, Namayandeh SM, Crockford J, Maroofian R, et al Digenic inheritance of mutations in the cardiac troponin (TNNT2) and cardiac β-myosin heavy chain (MYH7) as the cause of severe dilated cardiomyopathy. Eur J Med Genet 2017;60:485–488. 28642161 10.1016/j.ejmg.2017.06.008

[14] Bauersachs J, König T, van der Meer P, Petrie MC, Hilfiker-Kleiner D, Mbakwem A, et al Pathophysiology, diagnosis and management of peripartum cardiomyopathy: a position statement from the Heart Failure Association of the ESC Study Group on peripartum cardiomyopathy. Eur J Heart Fail 2019;21:827–843. 31243866 10.1002/ejhf.1493

[15] Morales A, Painter T, Li R, Siegfried JD, Li D, Norton N, et al Rare variant mutations in pregnancy-associated or peripartum cardiomyopathy. Circulation 2010;121:2176–2182. 20458009 10.1161/CIRCULATIONAHA.109.931220 PMC2900861

